# Phenylalkylammonium passivation enables perovskite light emitting diodes with record high-radiance operational lifetime: the chain length matters

**DOI:** 10.1038/s41467-021-20970-6

**Published:** 2021-01-28

**Authors:** Yuwei Guo, Sofia Apergi, Nan Li, Mengyu Chen, Chunyang Yin, Zhongcheng Yuan, Feng Gao, Fangyan Xie, Geert Brocks, Shuxia Tao, Ni Zhao

**Affiliations:** 1 Department of Electronic Engineering, The Chinese University of Hong Kong, Hong Kong SAR, People’s Republic of China; 2 Materials Simulation and Modelling, Department of Applied Physics, Eindhoven University of Technology, Eindhoven, The Netherlands; 3 Center for Computational Energy Research, Department of Applied Physics, Eindhoven University of Technology, Eindhoven, The Netherlands; 4 School of Electronic Science and Engineering, Xiamen University, Xiamen, People’s Republic of China; 5 Biomolecular and Organic Electronics, Linköping University, Linköping, Sweden; 6 Instrumental Analysis and Research Centre, Sun Yat-sen University, Guangzhou, People’s Republic of China; 7 Computational Materials Science, Faculty of Science and Technology and MESA+ Institute for Nanotechnology, University of Twente, Enschede, The Netherlands

**Keywords:** Electronic devices, Organic LEDs

## Abstract

Perovskite light emitting diodes suffer from poor operational stability, exhibiting a rapid decay of external quantum efficiency within minutes to hours after turn-on. To address this issue, we explore surface treatment of perovskite films with phenylalkylammonium iodide molecules of varying alkyl chain lengths. Combining experimental characterization and theoretical modelling, we show that these molecules stabilize the perovskite through suppression of iodide ion migration. The stabilization effect is enhanced with increasing chain length due to the stronger binding of the molecules with the perovskite surface, as well as the increased steric hindrance to reconfiguration for accommodating ion migration. The passivation also reduces the surface defects, resulting in a high radiance and delayed roll-off of external quantum efficiency. Using the optimized passivation molecule, phenylpropylammonium iodide, we achieve devices with an efficiency of 17.5%, a radiance of 1282.8 W sr^−1^ m^−2^ and a record *T*_50_ half-lifetime of 130 h under 100 mA cm^−2^.

## Abstract (web summary)

Perovskite light emitting diodes suffer from operational stability, showing rapid decay of performance within minutes to hours after turn-on. Here, the authors investigate how the steric and Coulomb interaction of ammonium passivation molecules with varying alkyl chain length can improve device stability by suppressing iodide ion migration.

## Introduction

In the past few years, the technology of perovskite light emitting diodes (PeLEDs) has developed rapidly, exhibiting highly attractive properties such as high efficiency, high luminance and radiance, excellent color tunability and purity, etc.^1–5^. The rapid advancement of PeLEDs is partially owing to the extensive material design and processing experience gained from the perovskite solar cell research^6^. However, in addition to the stability issues present in solar cells, there are several unique challenges associated with PeLEDs, such as the imbalanced charge injection, shunts associated with the very thin film thickness, and the rapid degradation of electroluminescence during operation^7–9^. In particular, due to intrinsic defects^10^, ion migration^11,12^, a poor temperature tolerance^13,14^, and the film morphology^15,16^, most PeLEDs degrade quickly, within several minutes to several hours of operation/after turn-off, even under a low current density less than 10 mA cm^−2^ (ref. ^17,18^). Such poor operational stability is a major bottleneck for the commercialization of this technology.

Major research efforts have been made to improve the stability of PeLEDs. For instance, layered two-dimensional (2D) perovskites were incorporated in PeLEDs in consideration of their superior thermal and structural stability compared to the three-dimensional (3D) perovskites^19,20^. Although this strategy has led to a *T*_50_ (the time it takes for external quantum efficiency (EQE) to drop to half of its maximum) of 100 h under 25 mA cm^−2^ (ref. ^20^), the EQE of such devices is somewhat compromised due to the poor charge transport between the inorganic layers^21,22^. Moreover, due to the enlarged band gaps of the 2D perovskites, the emission wavelength of these LEDs cannot yet reach the infrared range. Mixed 2D/3D perovskites have also been introduced to improve the stability of PeLEDs while maintaining high device performance^23,24^, achieving an estimated *T*_50_ > 200 h under initial radiance of about 10 W sr^−1^ m^−2^. Recently, there have been a few studies aiming to directly enhance the stability of pure 3D PeLEDs. Li et al. provided direct experimental evidence for an ion-migration induced device degradation mechanism and demonstrated largely improved stability of 3D FAPbI_3_-based PeLEDs by simultaneous incorporation of Cs and Rb cations in the perovskite lattice^11^. Another approach is the passivation of the perovskite surface defects, by introducing additives in the precursor solution. For instance, Wang et. al. introduced trifluoroacetate anions in the precursor solution to passivate the surface defects of CsPbBr_3_ perovskite films and achieved a *T*_50_ of 250 h at a low luminance of 100 cd m^−2^ (ref. ^25^). Park et al. introduced an organic semiconducting additive (2,2ʹ,2″-(1,3,5-benzinetriyl)-tris(1-phenyl-1-H-benzimidazole), TPBi) in the precursor and proposed that a perovskite/organic core–shell structure is formed and accounts for the improvement of the stability^26^. Miao et al. introduced benzylamine in the precursor and achieved PeLEDs with a *T*_50_ of 23.7 h at a high current density of 100 mA cm^−2^ (ref. ^27^). In our previous study^28^, we found that the ion diffusion at the interface between the perovskite and its top neighboring organic hole-transporting layer plays a critical role in the degradation process of PeLEDs. It is worth noting that to date almost all surface passivation or ligand engineering research of PeLEDs focuses on defect passivation, instead of ion-blocking. And different from solar cells, the fabrication of the perovskite emissive layer in PeLEDs involves the use of largely excess organic halide salts, which induces additional sources of mobile ions in PeLEDs. More importantly, the electric-field across the very thin perovskite layer in a PeLED is much higher than that in a solar cell. Therefore, ion migration is a much more difficult challenge to address in the field of PeLEDs. Developing an effective surface passivation method to suppress the ion-migration and its subsequent device degradation is critical for improving the stability of 3D perovskite-based PeLEDs.

In this work, we explore the surface treatment of 3D perovskite films with phenylalkylammonium iodides. By systematically varying the alkyl chain length, we investigate how the steric and Coulomb interactions of the ammonium passivation molecules affect the stability and electronic properties of the perovskite films and devices. Combining X-ray photo-electron spectroscopy (XPS), time-of-flight secondary ion mass spectrometry (ToF-SIMS), and theoretical modeling, we show that the phenylalkylammonium groups can stabilize the perovskite lattice through suppression of iodide ion migration. Very interestingly, the stabilization effect is enhanced with increasing chain length due to the stronger bonding of the passivating molecule to the perovskite surface and the increased steric hindrance for diffusion of iodide (I^−^) on the perovskite surface. With the optimized passivation molecule, phenylpropylammonium iodide (PPAI), we achieve a record *T*_50_ lifetime of 130 h under 100 mA cm^−2^, together with an EQE of 17.5% and a record radiance of 1282.8 W sr^−1^ m^−2^ on glass substrates.

## Results

### Passivation effects on films

**Fig. 1:**
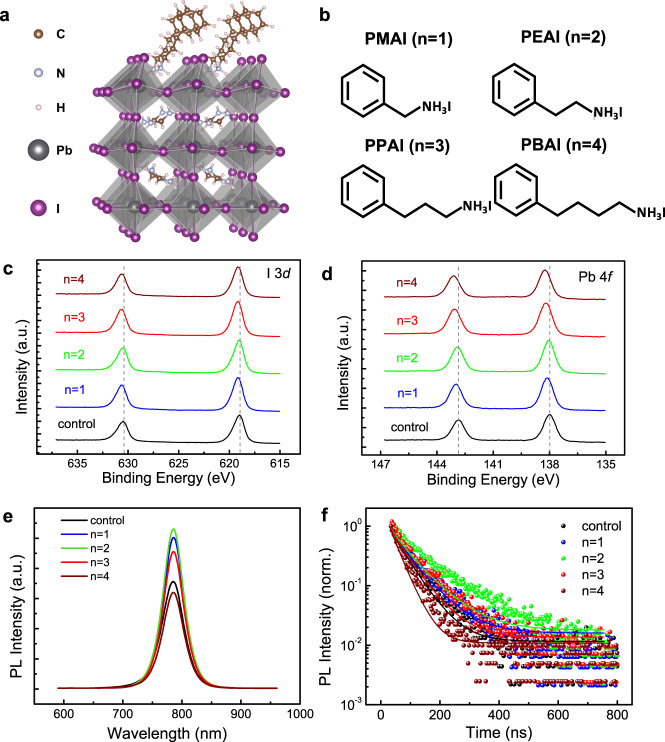
Perovskite structure and film characterizations. **a** Schematic diagram of a molecular passivated perovskite surface; **b** structures of the passivation molecules; **c** XPS I 3*d* spectra; **d** XPS Pb 4*f* spectra of the perovskite films without (control) and with passivation (*n* = 1–4); **e** PL spectra of the perovskite films without (control) and with passivation (*n* = 1–4) (excitation wavelength: 405 nm; excitation intensity: 70 mW cm^−2^); **f** TRPL data (solid circles) and fitting curves of the perovskite films without (control) and with passivation (*n* = 1–4) (excitation wavelength: 405 nm; excitation energy density: 0.3 μJ cm^−2^ pulse^−1^).

We use FA_0.83_Cs_0.17_PbI_3_ as a model composition for 3D perovskites. After the formation of the perovskite film, a surface treatment by phenylalkylammonium iodide is applied by spin-coating of the iodide salt solution (in chloroform) on the perovskite film, followed by an annealing process to remove the solvent (see “Methods” for the detailed fabrication process). Figure 1a illustrates the schematic diagram of the phenylalkylammonium-passivated perovskite lattice. In order to understand how the steric and Coulomb interactions of the molecules affect the passivation results, we investigate four molecular cations with the same end groups but different alkyl chain lengths, namely phenylmethylammonium iodide (PMAI, *n* = 1), phenethylammonium iodide (PEAI, *n* = 2), PPAI (*n* = 3), and phenylbutanammonium iodide (PBAI, *n* = 4), as shown in Fig. 1b.

The presence of the passivation layer and its bonding to the perovskite lattice are confirmed by the contact angle measurement (Supplementary Fig. 1), which reveals an increasing contact angle as the chain length increases, and XPS. As shown in the XPS spectra (Fig. 1c, d), the binding energies corresponding to the I 3*d* and Pb 4*d* core levels of the passivated samples all slightly shift to higher values compared to the control sample, an indication of enhanced binding of the iodide and lead ions on the perovskite surface^29^. We also note that the shifts are more pronounced in the *n* = 3 and *n* = 4 samples. (The comparison between the I 3*d* spectra of the pure passivation molecules and the passivated perovskites (shown in Supplementary Fig. 2) confirms that high-energy shift of the passivated films originates from the interactions between the passivation molecules and the perovskite lattice rather than the passivation molecules themselves.) Apart from the surface interaction, the morphology of the perovskite films shows no obvious change after the surface treatment (Supplementary Fig. 3) which is different from the impact introduced by passivating polymers^30^. The X-ray diffraction (XRD) data also suggest that the treatment does not change the crystallinity or the lattice structure of the perovskite films (Supplementary Fig. 4). Accordingly, the UV–Vis absorption spectra of the perovskite films remain almost unchanged (Supplementary Fig. 5) after the treatment. It is worth noting that no XRD signals corresponding to 2D perovskite phases are observed, although we cannot exclude that there might be an ultrathin layer of 2D phase present at the 3D perovskite surface.

We then examine the electronic properties of the perovskite films before and after surface treatment by ultraviolet photoelectron spectroscopy (UPS) and photoluminescence (PL) measurements. The UPS result shown in Supplementary Fig. 6 suggests that the treatment slightly raises the energy levels of the perovskite by 0.1 eV–0.3 eV. The treatment also enhances the PL intensity of the perovskite films except for the *n* = 4 case (Fig. 1e). Through the time-resolved photoluminescence (TRPL) measurements (Fig. 1f and Supplementary Fig. 7), we observe a consistent trend that surface treatments by PMAI (*n* = 1), PEAI (*n* = 2), and PPAI (*n* = 3) all increase the carrier lifetime in the perovskite films while the treatment by PBAI (*n* = 4) shortens the carrier lifetime. (Note that the steady-state PL is performed with a lower excitation intensity compared to TRPL and therefore reveals a more pronounced difference among the samples.) The TRPL data can be well fitted assuming a combination of monomolecular recombination *k*_1_ (trap-assisted recombination), bimolecular recombination *k*_2_ (band-to-band recombination), and Auger recombination *k*_3_ (refs. ^31,32^) (fitting parameters shown in Supplementary Table 1). In general, the PL measurements suggest that (1) surface treatment by PMAI (*n* = 1), PEAI (*n* = 2), or PPAI (*n* = 3) can all reduce non-radiative recombination by passivating the surface defects of the perovskite films, corresponding to reduced *k*_1_ values and that (2) surface treatment by PBAI (*n* = 4) slightly suppresses PL, possibly due to a low anchoring density (i.e., the density of the molecules that are effectively bonded to the perovskite surface) of the passivation molecules and/or the treatment causing formation of new recombination centers that accelerate non-radiative recombination.

### Passivation effects on devices

**Fig. 2:**
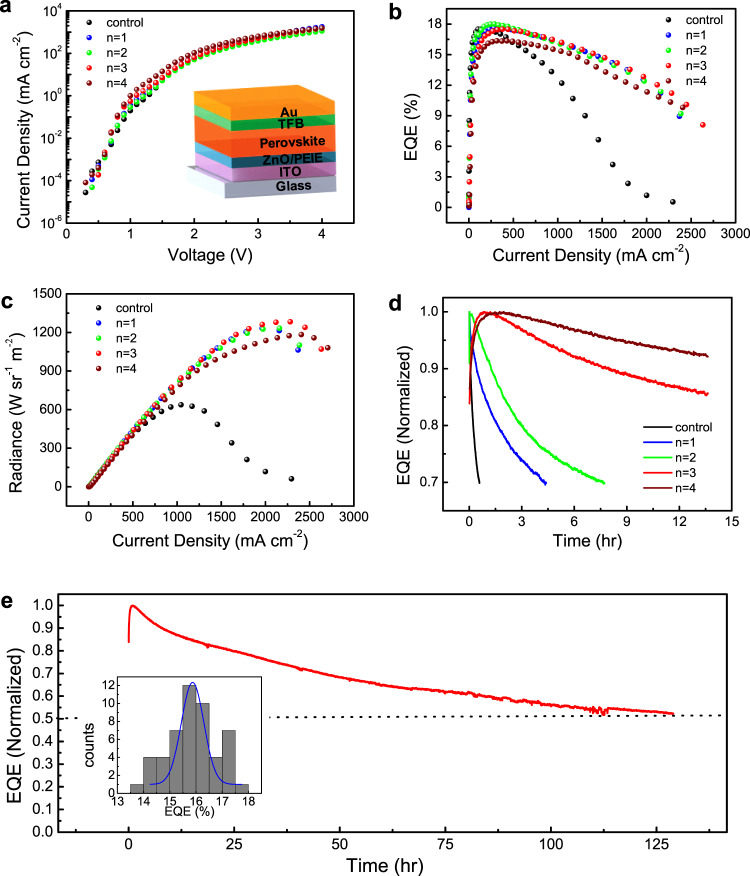
PeLED performance characterizations. **a** *J*–*V* curves for the control and treated devices; **b** EQE as a function of current density for the control and treated devices; **c** radiance as a function of current density for the control and treated devices; **d** stability test under a constant bias current density of 100 mA cm^−2^ for the control and treated devices; **e** *T*_50_ measurement under a constant current density of 100 mA cm^−2^ and histogram of peak EQE (inset) for the PPAI (*n* = 3)-treated PeLEDs.

In order to examine the effect of the surface treatment on the PeLED performance, we fabricate PeLED devices with a structure of gold (Au)/molybdenum oxide (MoO_3_)/poly(9,9-dioctylflu-16orene-co-*N*-(4-butylphenyl)diphenylamine) (TFB)/passivation molecule-perovskite/polyethyleneimine (PEIE)/zinc oxide (ZnO)/indium tin oxide (ITO). In this structure, TFB works as the hole-transporting layer and PEIE-modified ZnO works as the electron-transporting layer. The performances of the devices with and without passivation are characterized and illustrated in Fig. 2. It can be seen that except for *n* = 4, all devices show similar peak EQE (Fig. 2a) and electroluminescence peak (Supplementary Fig. 8); however, the roll-off of the EQE in the passivated devices is much less severe than that of the untreated (control) device. For the PBAI (*n* = 4)-treated sample, a reduced EQE is observed, which is consistent with the PL measurement results.

In contrast to the moderate variation in EQE, the operational stability of the PeLEDs is changed drastically after the surface treatment. Figure 2d shows the EQE decay of the control and passivated devices during a stability test under a constant biasing current density of 100 mA cm^−2^. The stability of all passivated devices is substantially enhanced. It is also noted that the efficiency of the PeLEDs exhibits an initial rise before the prolonged decay process starts. This phenomenon is commonly observed in PeLEDs^13,33–36^ and is so far speculated to be related to ion migration^36^ or trap filling/defect annihilation^37^. Note that the time scale of this initial rise is much longer than the step size of the EQE measurements; therefore this rise behavior does not affect the accuracy of the EQE measurements. More importantly, the enhancement of the operation lifetime depends highly on the value of *n*. The underlying mechanism for such strong chain length-dependence will be discussed in the next section.

Based on our overall evaluation of the device performance and stability, we propose PPAI (*n* = 3) as the optimal passivation molecule, among the ones studied here. The champion device with the PPAI treatment exhibits a peak EQE of 17.5%, a record high radiance of 1282.8 W sr^−1^ m^−2^ achieved on glass substrates (Fig. 2a–c). The histogram of the peak EQE based on 50 individual devices is shown in Fig. 2e, suggesting an average EQE of 15.87%. A long-term stability test under a constant current density of 100 mA cm^−2^ suggests a remarkable *T*_50_ lifetime of 130 h at high brightness (Fig. 2e). Table 1 provides a detailed comparison on the stability and performances of our device and other state-of-the-art PeLEDs with either high brightness or good stability. It can be seen that the PPAI-treated device represents the most stable high-radiance PeLEDs reported to date.

**Table 1:** Stability and performance comparison of PeLEDs.

Emission layer	Emission peak (nm)	Maxmium EQE	Maximum radiance (W sr^−1^ m^−2^)	Maximum luminance (cd m^−2^)	Operational current density (mA cm^−2^)	*T*_50_ stability (h)	Reference
FA_0.83_Cs_0.17_PbI_3_	789	17.50%	1282.7	—	100	130	This work
FAPbI_3_	802	14.20%	241	—	100	23.7	^27^
FAPbI_3_	803	20.70%	390	—	100	20	^33^
FAPbI_3_	780	19.60%	301.8	—	20	7	^51^
FAPbI_3_	800	21.60%	308	—	25	20	^52^
FAPbI_3_	802	17.30%	∼200	—	20	100	^34^
FAPbI_3_-DJ	785	5.20%	88.5	—	25	100	^20^
Cs_10_(MA_0.17_FA_0.83_)_(100-*x*)_PbBr_0.33_I_2.67_	650	8.21%	—	8777	1.76	~490	^53^
CsPbI_2.8_Br_0.2_	689	18.6%	—	—	5	20	^54^
FA_0.33_Cs_0.67_Pb(I_0.7_Br_0.3_)_3_	694	20.9%	—	<500	2.5	14	^55^
Cs_10_(MA_0.17_FA_0.83_)_(100-*x*)_PbBr_2.97_I_0.03_	569	12.98%	—	55370	1.42	~70	^53^
CsPbBr_3_ QD	520	21.63%	—	41900	<10	180.1	^36^
CsPbBr_3_-MABr	525	20.30%	—	3400	<10	104.56	^35^
CsPbBr_3_-KBr	520	7.70%	—	120000	<10	3	^13^
CsPbBr_3_	518	10.50%	—	16436	<10	250	^25^
PEA_2_Cs_1.6_MA_0.4_Pb_3_Br_10_	489	1.30%	—	5141	50	0.834	^16^
PEA_2_Cs_1.6_MA_0.4_Pb_3_Br_10_	479	5.20%	—	468	<10	1.5	^16^

### Mechanism of stability improvement

Based on the stability behavior, in the following, we categorize the devices into three groups: (1) untreated, (2) treated with a short alkyl chain ammonium (*n* = 1 and *n* = 2), and (3) treated with a long alkyl chain ammonium (*n* = 3 and *n* = 4). We focus on the comparison of the control, *n* = 1 (representing Group 2), and *n* = 3 (representing Group 3) samples to investigate the mechanism of the surface treatment-induced stability enhancement.

**Fig. 3:**
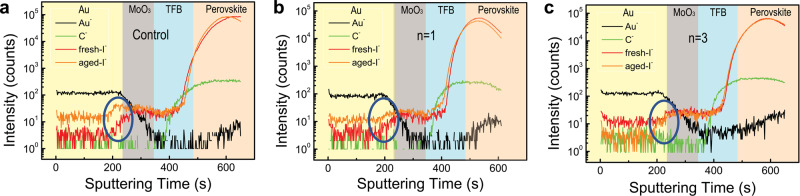
ToF-SIMS measurement results. ToF-SIMS of fresh device and aged device for **a** control sample, **b** PMAI (*n* = 1)-passivated device, **c** PPAI (*n* = 3)-passivated device. The blue circle highlights the local accumulation of iodide (I^−^) ions close to gold (Au).

As ion migration is found to be a major cause of degradation for PeLEDs^38,39^, we first perform ToF-SIMS to examine the ion distribution in the devices before and after bias stressing (stress current density: 100 mA cm^−2^; stress time: 16 h). In the control sample (Fig. 3a and Supplementary Fig. 9a), a clear accumulation of iodide ions close to gold is found after bias stressing, revealing severe iodide ion migration during device operation. Such behavior is partially suppressed in the PMAI (*n* = 1)-treated device (Fig. 3b and Supplementary Fig. 9b), while for the PPAI (*n* = 3)-treated device, we observe a complete suppression of this behavior, with the fresh and aged devices showing almost identical iodide distribution profiles (Fig. 3c and Supplementary Fig. 9c).

**Fig. 4:**
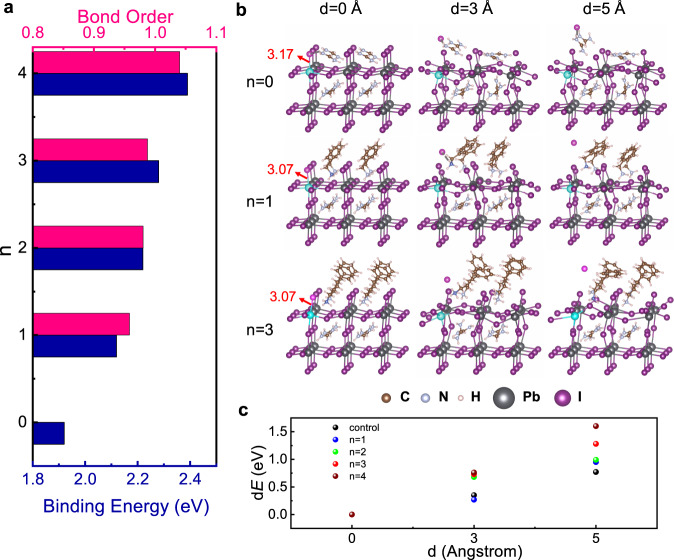
DFT analysis of perovskite surface. DFT analysis of perovskite surfaces passivated by molecules of *n* = 0 (reference FAPbI_3_) and *n* = 1 to *n* = 4. **a** Binding energy and bond order of the passivation molecules on the FAPbI_3_ perovskite surface. **b** Schematic pathway of iodide migration on the passivated perovskite surfaces. From left to right are configurations with zero iodide displacement (i.e., iodide ions are bonded in the lattice) and iodide displacement to distances of 3 and 5 Å away from the perovskite surface, respectively. From top to bottom are systems with molecules of *n* = 0 (reference FAPbI_3_), *n* = 1, and *n* = 3, respectively. The mobile iodide and the most affected species, such as, the surface lead and the ammonium group (the nitrogen) of the molecule are highlighted in colors pink, cyan, and blue, respectively. **c** Relative energies along the iodide diffusion path for molecules with *n* = 0, 1–4.

Our ToF-SIMS results suggest that the length of the alkyl chain plays an important role in blocking the ion migration. In order to gain an atomistic insight, we perform density functional theory (DFT) calculations to investigate the interactions between the passivation molecules (with alkyl chain *n* = 1–4) and the perovskite surface. First, we determine the adsorption geometry of phenylalkylammonium iodide passivation molecules with alkyl chain lengths from *n* = 1 to *n* = 4. We use the most stable Pb–I terminated perovskite surface and then investigate three different adsorption geometries for each molecule (Supplementary Fig. 10). We conclude that in all cases the most favorable geometry is similar to those depicted in Fig. 4b (the left row). Specifically, the iodide anion adsorbs on top of Pb, while the NH_3_ group of the molecular cations points to the corner Pb atom and forms hydrogen bonds with the surface iodide ions. The alkyl chain extends in the direction normal to the perovskite surface with the benzene ring pointing out of plane.

Having determined the adsorption geometry, we proceed with calculating the binding energy of the passivation molecules with alkyl chain *n* = 1–4 on the perovskite surface. The binding energy *E*_b_, calculated as *E*_b_ = | *E*_FAPbI3_ + *E*_mol_ *–* *E*_mol/FAPbI3_ | (1), is shown in Fig. 4a. We note that the formamidinium iodide (FAI)-terminated perovskite surface is also studied, where FAI binds to a Pb–I surface and we use this as a reference (*n* = 0). For all the examined values of *n*, the adsorption of the corresponding molecules on the perovskite surface is favorable, with binding energies ranging from 2.12 to 2.39 eV, considerably larger than the 1.92 eV of FAI (*n* = 0). Moreover, it is evident that the binding energy increases with increasing *n*, which indicates a stronger interaction between the larger molecules and the perovskite. The trend agrees well with the XPS results. This observation is further validated by the sum of bond orders, between the molecules and the perovskite (Fig. 4a). From Supplementary Table 2, we can conclude that the main difference in the binding strength comes from the hydrogen bonds between the NH_3_ group and I^−^ ions. With increasing *n*, the bond order of these H–I bonds increases (with the rest being almost constant), which geometrically is demonstrated by shorter H–I bond length (left column in Fig. 4b).

Next, we study the role of the passivation molecules in suppressing the iodide migration. We built an iodide diffusion pathway by moving one iodide ion away, step-by-step, from the perovskite surface. Figure 4c summarizes the relative energies of all configurations along the iodide diffusion path for each molecule (*n* = 0–4), and Fig. 4b shows the corresponding molecular configurations for *n* = 0, *n* = 1, and *n* = 3. The energy landscapes clearly show that larger molecules make the extraction of I^−^ from the perovskite surface much more difficult. Careful investigation of the positions of the species near the moving I^−^ (Fig. 4b) reveals an interesting effect: the NH_3_ group tends to move along with the migrating I^−^ because of the strong hydrogen bond formed between the two (I–H). This is especially evident in the case of a moderate displacement of I^−^, e.g. at 3 Å. However, molecules with shorter chains (*n* = 0 or *n* = 1) are much easier to rotate due to smaller steric hindrance, and they can more easily reposition after the extraction of an I^−^ ion, as can be confirmed with the newly formed hydrogen bonds between the molecules and I^−^ at relatively short distances of less than 2.7 Å (Supplementary Fig. 11). The repositioning leads to (new) lowest energy states. It is worth noting that the enhanced hydrogen bond between the NH_3_ group of the passivation molecules and the I^−^ ion subsequently pulled the I^−^ ion to a closer distance to the surface Pb ion. From the calculation results shown in Fig. 4b and Supplementary Table 3, one can see that the Pb–I bond is shortened from 3.17 Å (for an unpassivated surface) to 3.07 Å (for a passivated surface). Accordingly the bond order is increased from 0.46 to 0.53 (Supplementary Table 4), suggesting a stronger Pb–I bond. This observation agrees well with the XPS results, where simultaneous shifts to higher energy values were found for the core levels of both I and Pb. In contrast, such repositioning is not feasible for larger molecules due to a larger steric hindrance; therefore, they retain a similar position before and after the extraction of I^−^. This effect becomes more dominant when I^−^ is placed further away from the surface, i.e. at 5 Å (as can be seen in the configurations without hydrogen bonds in Supplementary Fig. 11), leading to the largest energy for the longest molecule.

Altogether, our analysis indicates a strong binding of the passivation molecules and the perovskite surface via enhanced hydrogen bonds, and the layer of molecules acting as passivation agent to prevent the diffusion of the surface I^−^. More importantly, the strength of such passivation largely depends on the alkyl chain length: the longer the chain length, the larger the steric hindrance and thus the stronger the resistance to rotation and reposition to accommodate the migrating I^−^ ions. These results agree well with our experimental observation that the PPAI (*n* = 3) treatment is much more effective than the PMAI (*n* = 1) treatment in suppressing the iodide migration.

## Discussion

In summary, we explored surface passivation of 3D perovskite films with a series of phenylalkylammonium iodides of varying alkyl chain lengths. Combining experimental characterization and theoretical modeling, we show that the phenylalkylammonium groups can stabilize the perovskite surface through the suppression of iodide ion migration. More importantly, the stabilization effect is enhanced with increasing alkyl chain length, due to the stronger binding of the passivating molecule on the perovskite surface and the increased steric hindrance to reconfiguration required to accommodate I^−^ migration. Using the optimized passivation molecule, PPAI, we achieve PeLEDs with an EQE of 17.5%, a record radiance of 1282.8 W sr^−1^ m^−2^ on glass substrates, and a record *T*_50_ half-lifetime of 130 h under 100 mA cm^−2^. Our work highlights the importance of the chain length effect in suppressing the ion migration, revealing that a very small variation in the surface molecular structure can result in a remarkable improvement in device stability and sheds light on the design of new passivation molecules that could render highly efficient and stable perovskite LEDs.

## Methods

### Materials

Cesium iodide (CsI), FAI, and 5-ammonium valeric acid iodide (5AVAI) were purchased from Greatcellsolar. Lead iodide (PbI_2_) and PEAI (2-phenethylammonium iodide) were purchased from Tokyo Chemical Industry Co. LTD. PMAI, PPAI, and PBAI were bought from Xi’an Polymer Light Technology Corp. ZnO nanocrystals were synthesized according to the published recipe^40^. The other chemicals were purchased from Sigma-Aldrich.

### Device fabrication

ITO substrate was cleaned in Hellmanex water solution by sonication for 30 min, and subsequently cleaned with deionized water, acetone, isopropanol in sequence by sonication for 15 min, and treated by UV-Ozone for 15 min before use. Then, the ZnO layer was spun on the substrate at 6000 r.p.m. for 40 s and annealed at 150 °C for 30 min. PEIE (3.9 mg mL^−1^ in 2-methoxyethanol) layer was spun on ZnO at 5000 r.p.m. for 40 s and annealed at 90 °C for 10 min to modify the work function of ZnO and enhance the wettability. Then a 0.15 mmol mL^−1^ perovskite precursor (*n*(CsI):*n*(FAI):*n*(PbI_2_):*n*(Pb(SCN)_2_):*n*(5AVAI)) = 0.17:1.23:0.8:0.2:0.1) was spun on the substrates at 500 r.p.m. for 3 s and 8000 r.p.m. for 45 s, respectively. The 120 μL chlorobenzene was dripped on the substrate after 16 s, and then the film was annealed at 100 °C for 15 min. For the surface treatment, 0.5 mg molecules were added in 1 mL chloroform and the solution was filtered before diluting the solution with threefold chloroform. Then the solution was deposited at 6000 r.p.m. for 30 s on the perovskite films and the treated film was annealed at 100 °C for 3 min to volatilize remnant solvent. After cooling down to room temperature, the TFB (15 mg mL^−1^ in m-xylene) layer was spun on perovskite layer at 2500 r.p.m. for 30 s. Finally, 7 nm of MoO_3_ and 50 nm of Au were evaporated on the top by thermal evaporator through a shadow mask defining an electrode area of 2.25 mm^2^.

### Characterization

The XRD was tested with a Rigaku ru-300 diffractometer (Cu Kα irradiation, *λ* = 1.5406 Å). The SEM test was performed by HR-FESEM, FEI, Quanta 400. The XPS was conducted by Al Kα X-ray gun (*hv* = 1486.6 eV) with a VG ESCALAB 220i-XL surface analysis system. For the characterization of device performance, the *J*–*V* curves, radiance, and EQE were all carried out in nitrogen-filled glovebox through a QE-Pro spectrometer coupled with a fiber integration sphere (FOIS-1) and a Keithley 2400 source meter. The TRPL was taken on an Edinburgh Instruments spectrometer (FLS1000) with a 405-nm pulsed laser (75 ps, 0.5 MHz). The instrument resolution was less than 20 ps with the response function (IRF) less than 130 ps. And the tested perovskite films were fabricated on the ZnO/PEIE substrate. The ToF-SIMS test was taken on Model TOF‐SIMS 5 with the pulsed primary ions of a Bi^+^ (30 keV, 1 pA) ion gun for the sputtering and an O^+^ pulsed secondary ion beam for the analysis (0.5 keV, 100 nA) with an analysis area of 96 µm × 96 µm. The stability test was taken on the EQE test platform and calibrated by commercialization system made by Guangzhou Jinghe Ltd.

### First-principles calculation

DFT calculations were performed using the Projector Augmented Wave (PAW) method as implemented in the Vienna Ab-Initio Simulation Package (VASP)^41–44^. The electronic exchange-correlation interaction was described by the functional of Perdew, Burke, and Ernzerhof (PBE) within the spin-polarized generalized gradient approximation (GGA)^45^. Energy and force convergence criteria of 10^−5^ eV and 10^−1^ eV Å^−1^, respectively, were used in all calculations. The D3 correction^46^ was employed to account for the van der Waals interactions due to the presence of the organic molecules.

For the geometry optimization and the calculation of the binding energies and the bond orders, 9-layer Pb–I-terminated FAPbI_3_ slabs were used (cubic unit cell with a fixed lattice parameter of *a* = 6.36 Å) and a vacuum region of ~30 Å separating the slabs. To model the passivation effect of the molecules, CH_5_(CH_2_)_*n*_NH_3_I molecules were put on top of the above-mentioned Pb–I-terminated slab. The calculations were performed with a 3 × 3 × 1 Γ-centered *k*-point grid and a kinetic energy cutoff of 500 eV. A dipole correction was employed to avoid interaction between periodic images^47^. During the geometry optimization, all the atoms were allowed to relax in a fixed cell.

The binding energies *E*_b_ of the passivation molecules on FAPbI_3_ were calculated as:

1 $$E_{\mathrm{b}} = \left| {E_{{\mathrm{FAPbI}}_3} + E_{{\mathrm{mol}}} - E_{{\mathrm{mol}}/{\mathrm{FAPbI}}_3}} \right|$$

where *E*_mol/FAPbI3_, *E*_FAPbI3_, and *E*_mol_ are the total energies of FAPbI_3_ with an adsorbed passivation molecule per surface unit cell, the clean FAPbI_3_ surface, and the CH_5_(CH_2_)_*n*_NH_3_I, respectively. For the calculation of the bond orders, the density-derived electrostatic and chemical (DDEC6) method was used^48–50^. The bond order is a measure of the strength of a covalent bond, where larger the bond order corresponds to stronger bond.

In order to study the effect of the passivation molecules on the migration of I ions, 2 × 2 supercells of the 9-layer slabs were used and optimized by using one *k*-point (Γ point). To determine the energies of several configurations with a migrating I ion, the top two layers of the FAPbI_3_ slab could relax, while the bottom seven layers and the position of the “migrating” I ions were fixed.

## Supplementary information

Supplementary Information

## Data Availability

The data that support the findings of this study are available from the corresponding authors upon reasonable request.
