# Supplementary material for: Phenylalkylammonium passivation enables perovskite light emitting diodes with record high-radiance operational lifetime: the chain length matters
Source: Nat Commun. 2021 Jan 28;12:644. doi: 10.1038/s41467-021-20970-6 (PMC7843623; doi:10.1038/s41467-021-20970-6)
Supplement: Supplementary file 1 — Supplementary Information [file 41467_2021_20970_MOESM1_ESM.pdf]

Supplementary Information

Phenylalkylammonium Passivation Enables Perovskite Light Emitting Diodes with Record High-radiance Operational Lifetime: The Chain Length Matters

Yuwei Guo[1], Sofia Apergi[2,3], Nan Li[1], Mengyu Chen[1,4], Chunyang Yin[5], Zhongcheng Yuan[5], Feng Gao[5], Fangyan Xie[6], Geert Brocks[2,3,7], Shuxia Tao[2,3]*, Ni Zhao[1]*

1 Department of Electronic Engineering, The Chinese University of Hong Kong, Shatin, N.T. HKSAR

2 Materials Simulation and Modelling, Department of Applied Physics, Eindhoven University of Technology, 5600MB Eindhoven, The Netherlands

3 Center for Computational Energy Research, Department of Applied Physics, Eindhoven University of Technology, 5600 MB Eindhoven, The Netherlands

4 School of Electronic Science and Engineering, Xiamen University, Xiamen 361005, P. R. China

5 Biomolecular and Organic Electronics, Linköping University, S-581 83, Linköping, Sweden

6 Instrumental Analysis and Research Centre, Sun Yat-sen University, Guangzhou 510275, P. R. China

7 Computational Materials Science, Faculty of Science and Technology and MESA+ Institute for Nanotechnology, University of Twente, P.O. Box 217, 7500 AE Enschede, The Netherlands

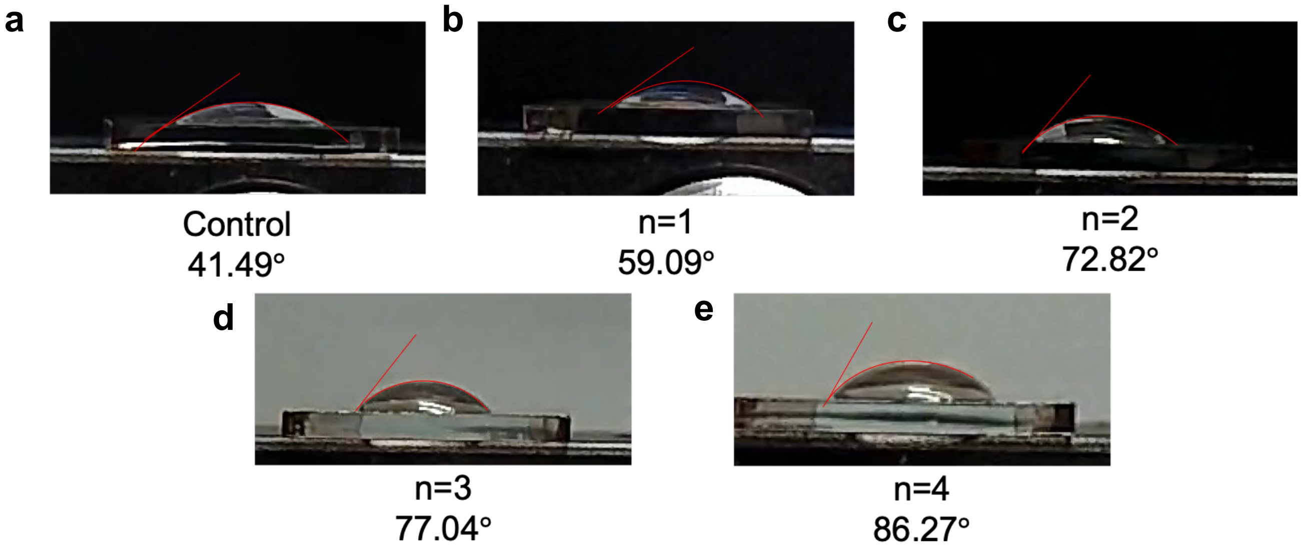

Supplementary Figure 1. Contact angle of **a**) control sample, **b**) PMAI (n=1), **c**) PEAI (n=2), **d**) PPAI (n=3), **e**) PBAI (n=4)

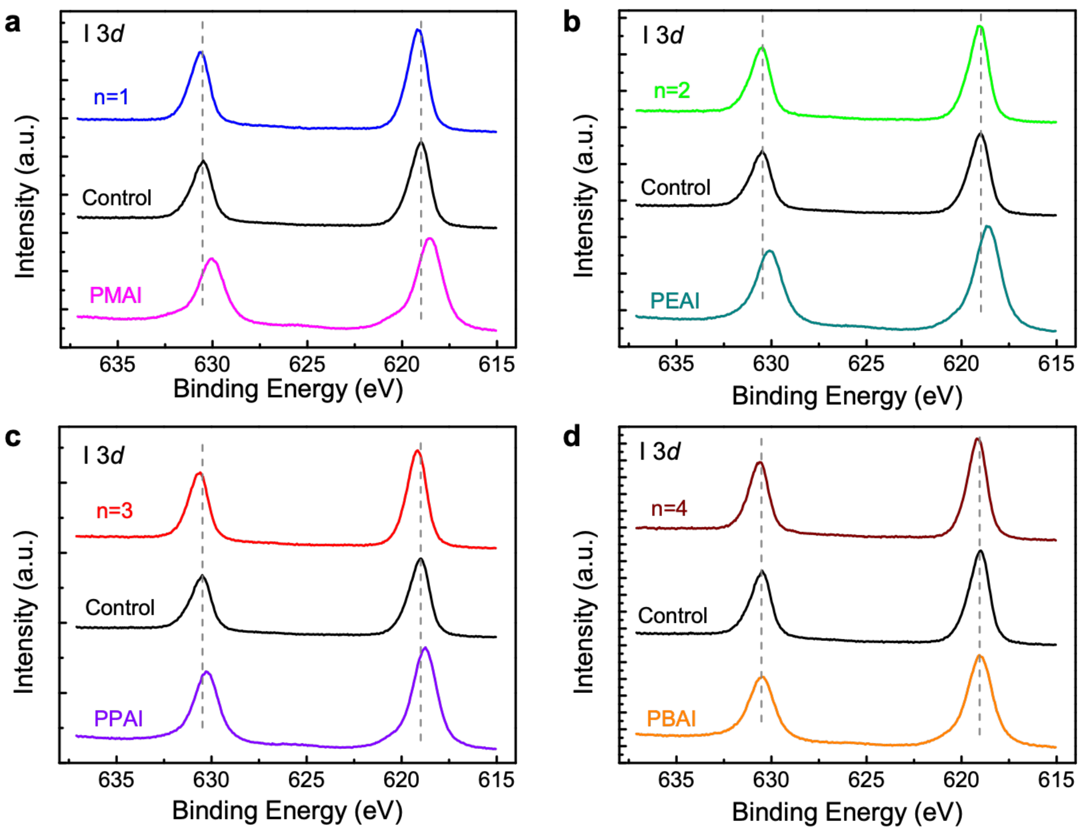

Supplementary Figure 2. XPS comparison among the pure molecule films (bottom curve), perovskite films without passivation (middle curve) and with passivation (top curve) for **a**) PMAI, **b**) PEAI, **c**) PPAI, and **d**) PBAI

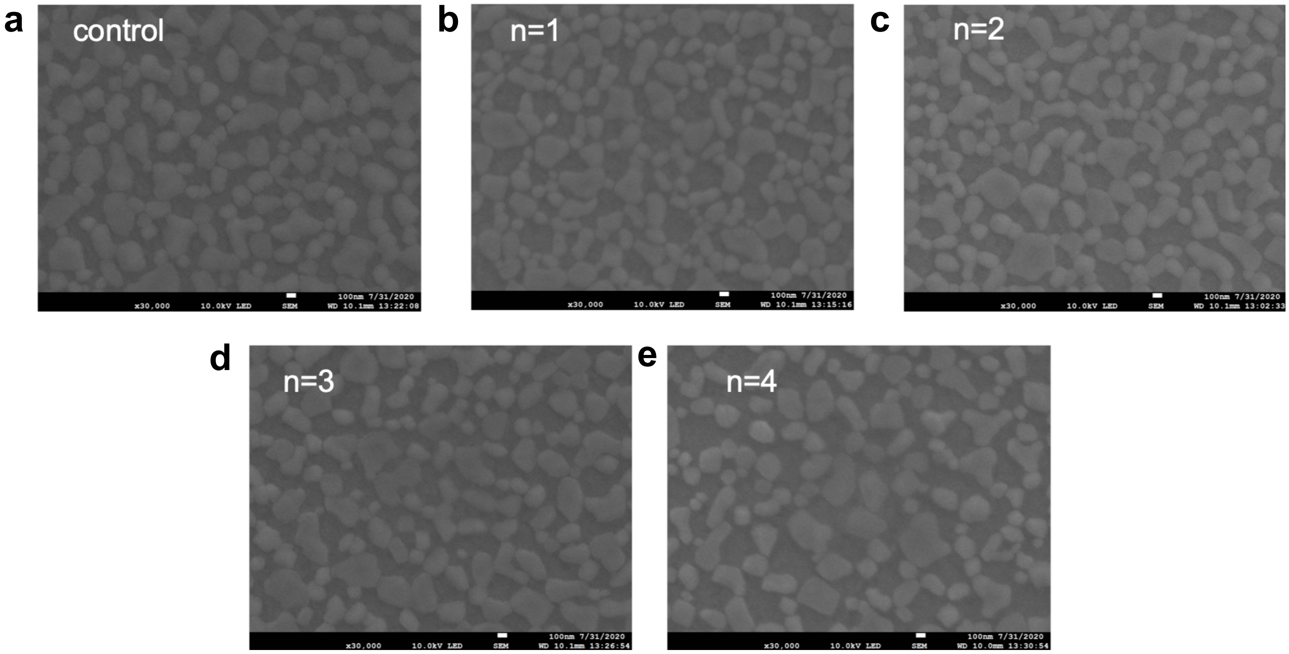

Supplementary Figure 3. SEM images of **a**) the control sample and the perovskite films treated with **b**) PMAI (n=1), **c**) PEAI (n=2), **d**) PPAI (n=3), **e**) PBAI (n=4)

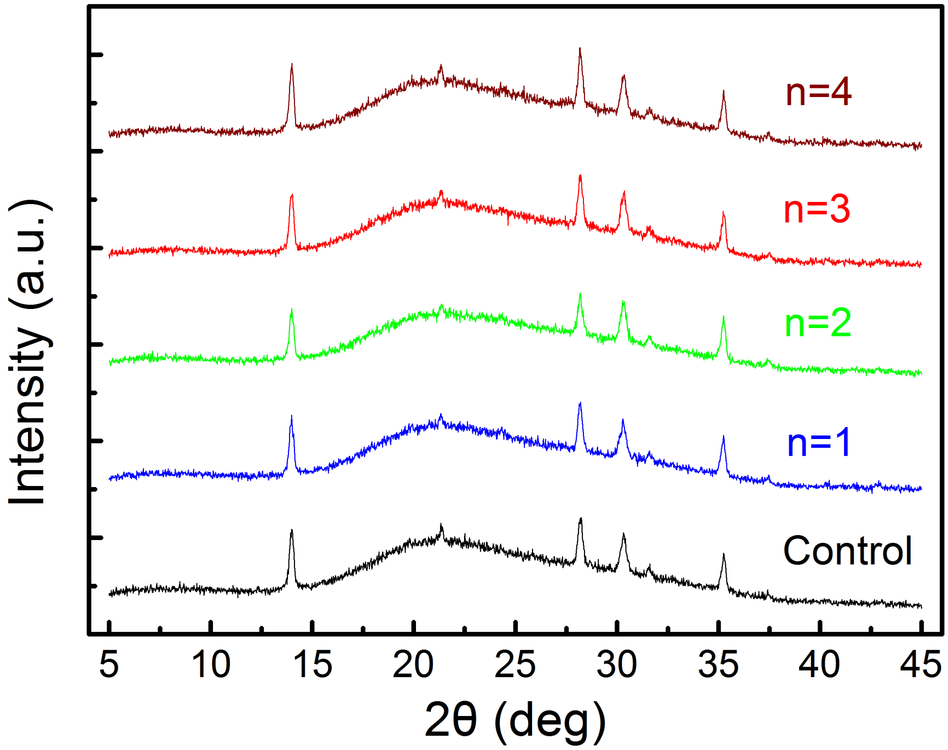

Supplementary Figure 4. XRD data comparison for the control sample and the perovskite films treated with PMAI (n=1), PEAI (n=2), PPAI (n=3), and PBAI (n=4)

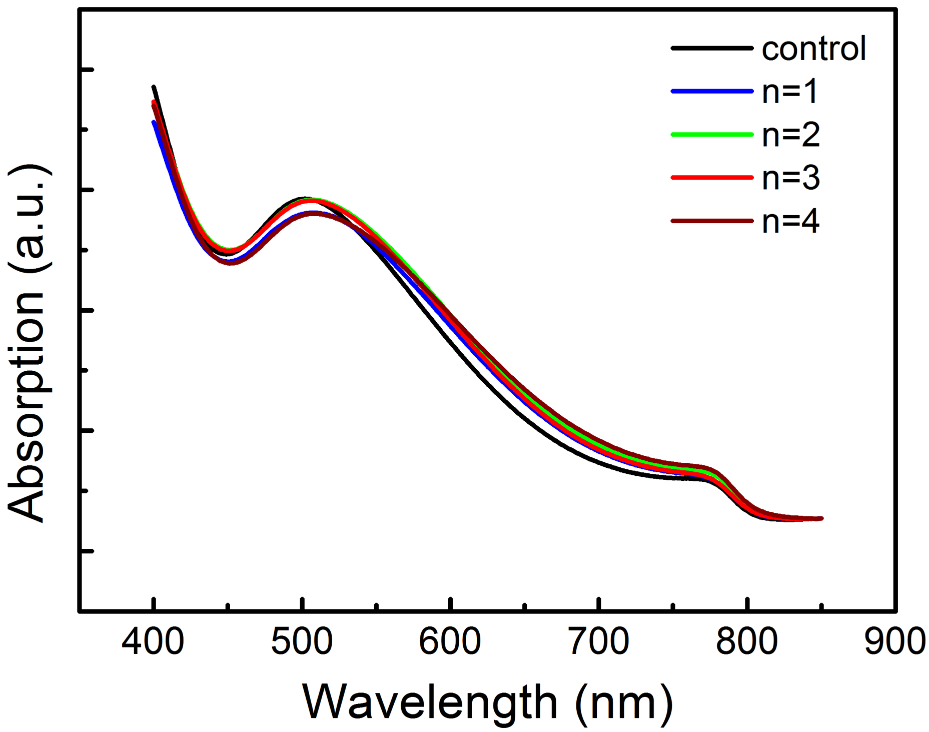

Supplementary Figure 5. Absorption spectra of the control sample and the perovskite films treated with PMAI (n=1), PEAI (n=2), PPAI (n=3), and PBAI (n=4)

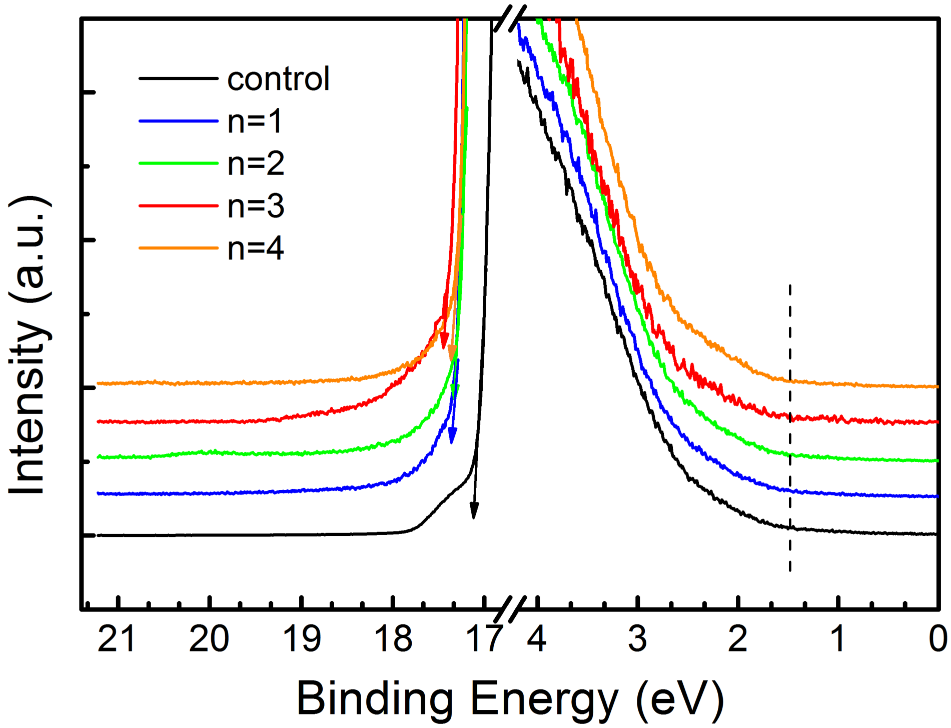

Supplementary Figure 6. UPS spectra of the control sample and the perovskite films treated with PMAI (n=1), PEAI (n=2), PPAI (n=3), and PBAI (n=4)

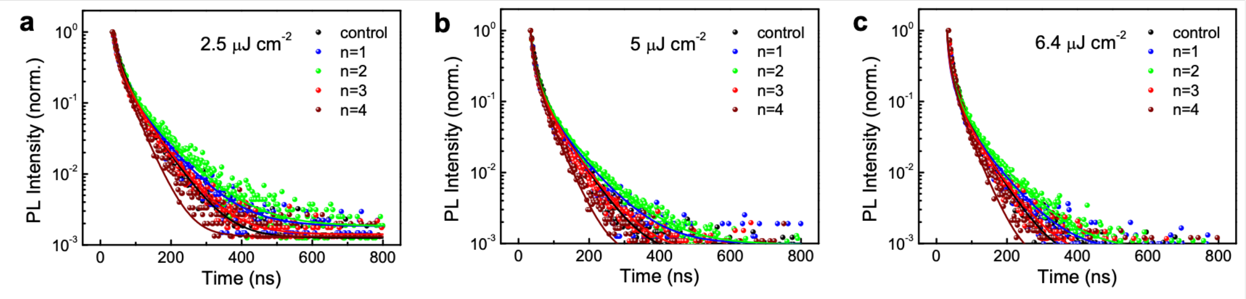

Supplementary Figure 7. Light-intensity-dependent time-resolved photoluminence data (solid circles) and fitting curves for the perovskite films with and without treatment under an excitation density of **a**) 2.5 μJ cm^{-2} pulse^{-1} **b**) 5 μJ cm^{-2} pulse^{-1} **c**) 6.4 μJ cm^{-2} pulse^{-1}

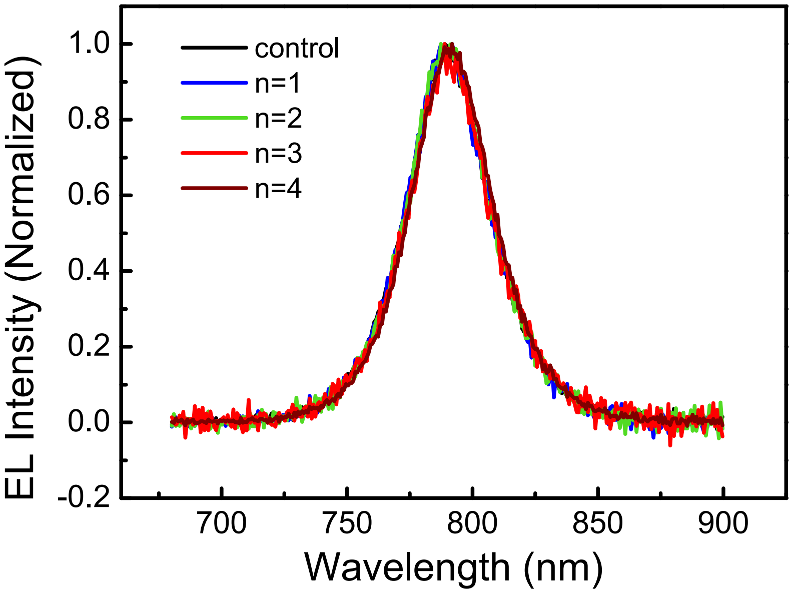

Supplementary Figure 8. EL spectra of the control sample and the perovskite films treated with PMAI (n=1), PEAI (n=2), PPAI (n=3), and PBAI (n=4)

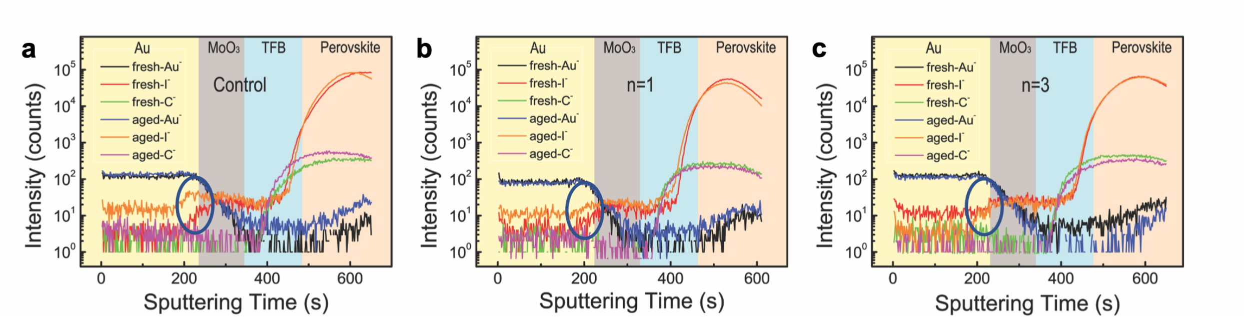

Supplementary Figure 9. ToF-SIMS of fresh device and aged device for **a**) control sample, **b**) PMAI (n=1) passivated device, **c**) PPAI (n=3) passivated device. The blue circle highlights the local accumulation of iodide (I^-) ions close to gold (Au).

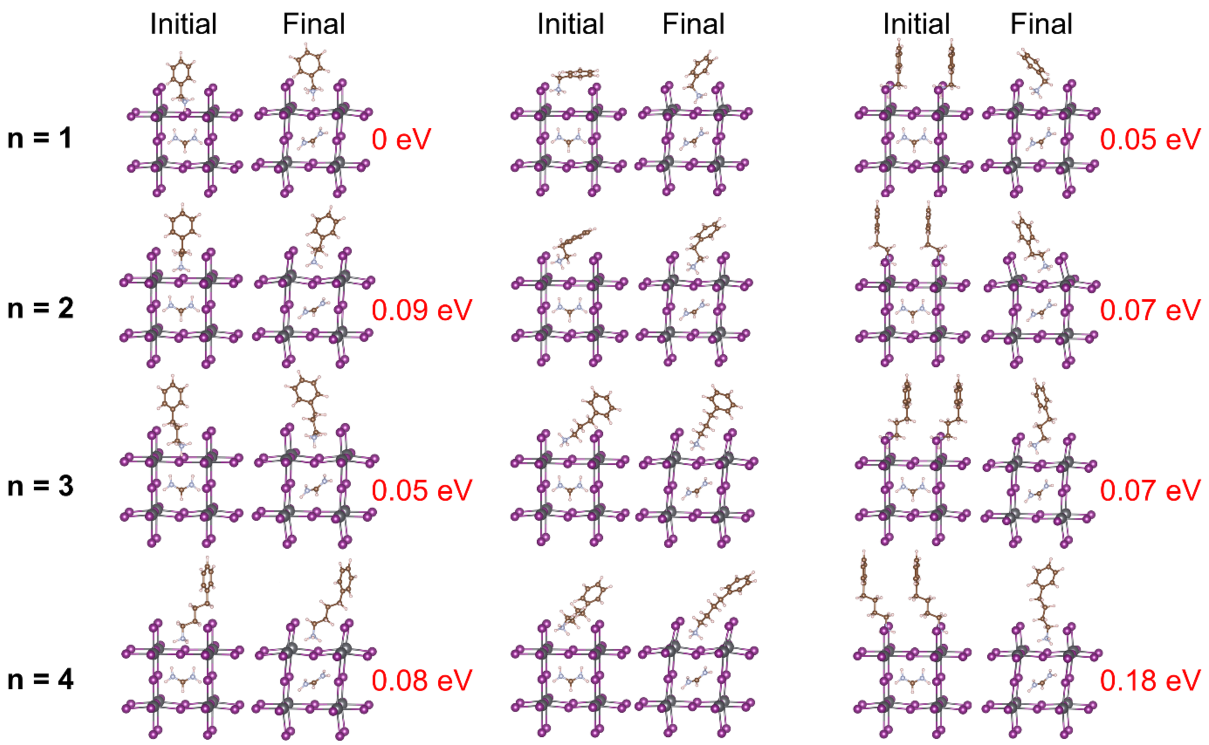

Supplementary Figure 10. Three sets of molecular configurations of the initial and relaxed (final) states, which were tested to determine the most favorable adsorption geometry for the passivation molecule on $FAPbI_3$. The configurations with the lowest energy are displayed in the center. On the left and right are the configurations with higher energies and in red is their energy difference from the most favorable structure (for a certain value of n).

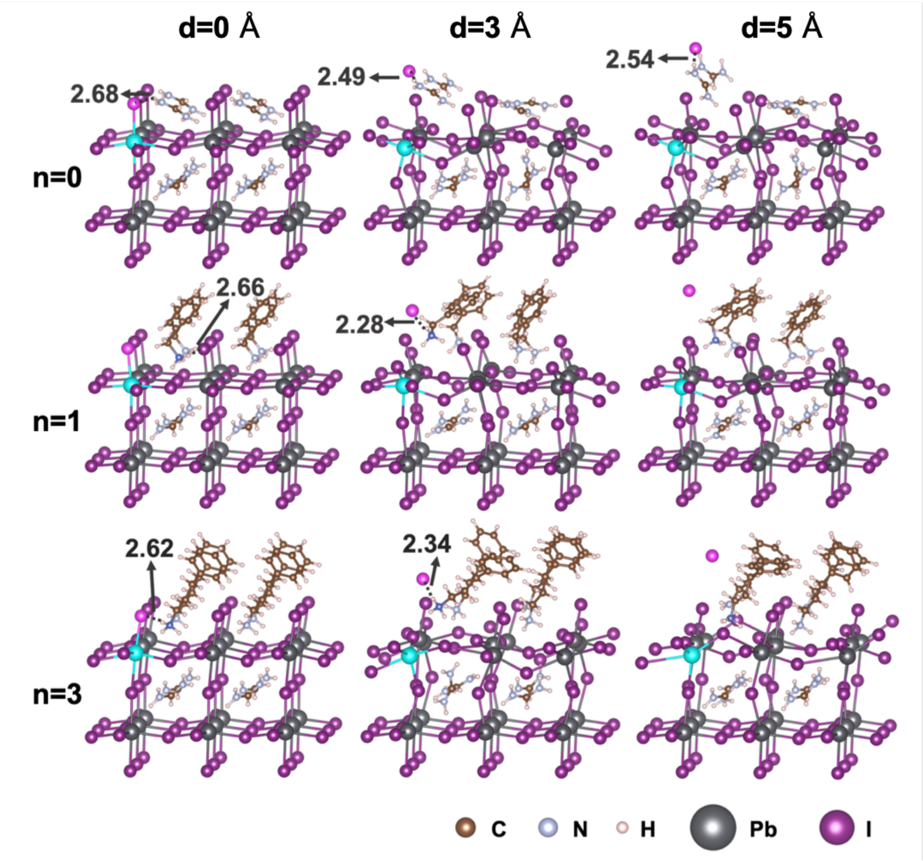

Supplementary Figure 11. Schematic pathway of iodide migration on the passivated perovskite surfaces. The numbers in the figure show the lengths of the hydrogen bonds

Supplementary Table 1. Fitting parameters for the light- intensity-dependent time-resolved photoluminence data based on the equation [1-2] $\frac{dn}{dt} = -k_1 \cdot n - k_2 \cdot n^2 - k_3 \cdot n^3 + G$ (1)

	k_1 (s^{-1})	Standard Error	k_2 (cm^3 s^{-1})	Standard Error	k_3 (cm^6 s^{-1})	Standard Error
control	1.66×10^{7}	8.20×10^{5}	1.43×10^{-10}	6.59×10^{-13}	1.04×10^{-28}	1.05×10^{-30}
n=1	1.24×10^{7}	9.85×10^{5}	2.06×10^{-10}	8.73×10^{-13}	1.63×10^{-28}	1.55×10^{-30}
n=2	1.09×10^{7}	9.01×10^{5}	2.09×10^{-10}	2.95×10^{-13}	1.36×10^{-28}	1.86×10^{-31}
n=3	1.48×10^{7}	9.33×10^{5}	1.82×10^{-10}	8.45×10^{-13}	1.54×10^{-28}	1.35×10^{-30}
n=4	2.54×10^{7}	7.94×10^{5}	1.48×10^{-10}	5.27×10^{-13}	8.15×10^{-29}	9.07×10^{-31}

Supplementary Table 2. Bond orders between the adsorbent and the perovskite, along with the contributions to the bond order from NH_3 and $C_6H_5(CH_2)_n$.

Adsorbent	Bond Order		
	Total	NH_3	$C_6H_5(CH_2)_n$
n = 1	0.96	0.49	0.47
n =2	0.98	0.52	0.46
n =3	0.99	0.52	0.46
n = 4	1.04	0.55	0.49
FA	0.93	-	-

Supplementary Table 3. Pb-I bond lengths in the surface Pb-I octahedron. The terminal I bond with Pb is significantly shorter, therefore stronger, in the case of the adsorbed passivation molecules, compared to the FA-I terminated perovskite.

Structure	x_1	x_2	y_1	y_2	z_1	z_2
Plain	3.18	3.18	3.17	3.2	3.06	-
n=1	3.19	3.18	3.18	3.2	3.34	3.07
n=2	3.17	3.22	3.18	3.19	3.38	3.09
n=3	3.14	3.27	3.19	3.18	3.38	3.07
n=4	3.13	3.28	3.19	3.19	3.35	3.1
FA-I	3.17	3.2	3.18	3.19	3.3	3.17

Supplementary Table 4. Pb-I bond order in the surface Pb-I octahedron. The Pb-I bond order is significantly larger (z_2 is the one formed between the passivation molecule and the surface Pb), therefore stronger, in the case of the adsorbed passivation molecules, compared to the FA-I terminated perovskite.

Structure	x_1	x_2	y_1	y_2	z_1	z_2
n=1	0.42	0.42	0.42	0.42	0.33	0.52
n=2	0.44	0.4	0.42	0.42	0.3	0.52
n=3	0.46	0.37	0.42	0.42	0.3	0.53
n=4	0.47	0.36	0.41	0.41	0.31	0.5
FA-I	0.44	0.41	0.42	0.41	0.35	0.46

Reference

1. Droseros, N. *et al*. Origin of the Enhanced Photoluminescence Quantum Yield in $MAPbBr_3$ Perovskite with Reduced Crystal Size. *ACS Energy Lett*. **3**, 1458–1466 (2018).
2. Xing, G. *et al*. Transcending the slow bimolecular recombination in lead-halide perovskites for electroluminescence. *Nat. Commun*. **8**, 14558 (2017).
